# Recent Development in Modeling of Coated Spherical Contact

**DOI:** 10.3390/ma13020460

**Published:** 2020-01-18

**Authors:** Zhou Chen, Izhak Etsion

**Affiliations:** Department of Mechanical Engineering, Technion-Israel Institute of Technology, Haifa 32000, Israel; chenzhouhit@gmail.com

**Keywords:** contact mechanics, coating, coated spherical contact, modeling

## Abstract

Since a coated rough surface can be modeled as a collection of many spherical coated asperities, in order to understand the coated rough surface contact, it is required to first model a single coated spherical contact. This review paper presents a comprehensive summary of the coated spherical contact modeling and its experimental validation that was done mostly by the authors’ group at the Technion and published in the relevant literature. The coated spherical contact is considered under two loading modes, namely pure normal loading and combined normal and tangential loading. Based on the normally loaded spherical contact results, a coated rough surface contact modeling is presented. In addition, experimental results that show an interesting correlation with the coated spherical modeling are briefly discussed. Finally, some limited work on the bilayer/multilayer coated spherical contact is introduced.

## 1. Introduction

Coatings are widely used in many applications to improve the tribological performance of contacting surfaces with relative sliding, e.g., [1,2,3,4,5,6]. Much effort is devoted to selecting a proper coating thickness in order to minimize friction and wear. However, this effort still consists mainly of experimental trial and error, which is expensive and time-consuming. Thus, an efficient scientific approach based on theoretical modeling is required. This problem is quite complex [7] involving various parameters such as mechanical properties of coating and substrate, surface roughness and mode of loading to name a few.

The contact of real rough surfaces can be treated by the ‘asperity-based model’ proposed by Greenwood and Williamson some 50 years ago [8]. According to this model, a rough surface is composed of multiple asperities having spherical tips at their summits and a statistical distribution of summit heights. Contact occurs at the summits of the highest asperities and it is assumed that these asperities are far enough from each other so that they do not interact. Hence, it is possible to incorporate the contact behavior of a single asperity in a statistical model of multiple asperities to study the contact mechanics of rough surfaces. Therefore, the contact mechanics of coated rough surfaces should start with modeling of the contact behavior of a single coated asperity, i.e., coated spherical contact.

Most of the studies done so far on coated spherical contact are based on indentation. One purpose of this approach is to characterize mechanical properties of the coating, e.g., [9,10,11,12]. In these studies, a hard spherical tip indents a coated flat to obtain the relation between the load and indentation depth. Another purpose is to characterize the tribological properties of coated surfaces, e.g., [13,14,15,16]. This is usually done by using a spherical indenter to slide over coated surfaces. However, when the indenter is sliding over coated surfaces, the plowing effect results in abrasive wear that often causes unexpectedly high wear rate. Fortunately, abrasive wear can be easily eliminated by various means as opposed to adhesive wear that is unavoidable [17]. Hence, the latter case is of greater importance for good tribological design. For this reason, the flattening of a coated sphere, rather than the indentation, is of greater interest.

**Figure 1:**
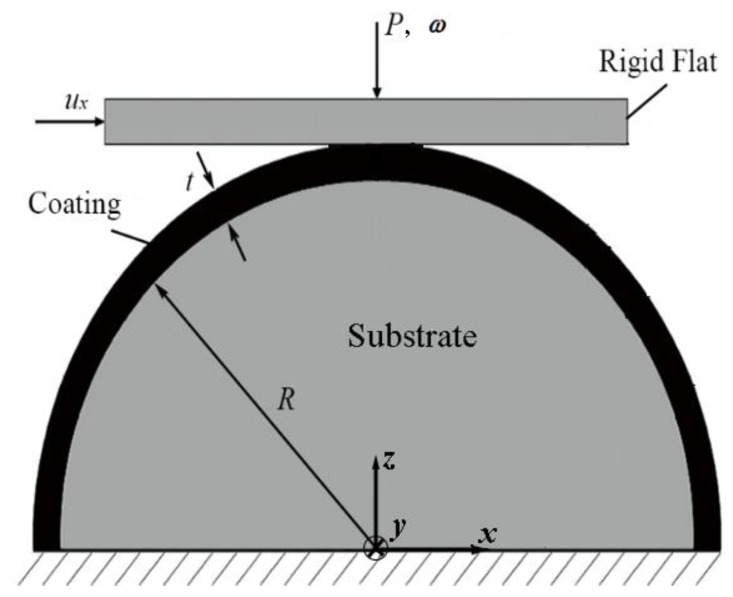
A model for the flattening of a coated spherical asperity, where a deformable coated sphere is in contact with a rigid flat.

Figure 1 schematically shows the flattening case of a coated spherical contact where the coated sphere is composed of a spherical substrate of radius *R* and coating of thickness *t*. When the coating and substrate materials are identical, the coated spherical contact reduces to the simpler homogeneous spherical contact without coating. This fundamental problem of contact mechanics was first solved in 1882 by Hertz (see, e.g., [18]) for the elastic regime of normal loading. A normal contact load, *P*, results in an interference *ω*, (deformation of the sphere summit), and a contact area *A*.

(1) $$P=\frac{4}{3}\frac{ER^{1/2}\omega^{3/2}}{\left(1-\upsilon^{2}\right)},$$

(2) A=πωR,

where *E* and *υ* are Young’s modulus and Poisson’s ratio, respectively of the sphere material.

The pioneering Hertz solution was extended in 1987 into the elastic-plastic regime [19]. By combining normal and tangential loading, sliding inception and static friction were studied for the uncoated spherical contact, e.g., [20,21], and interesting results regarding the effect of normal load on the static friction coefficient were found. Extensive research work on elastic–plastic contact can be found in the literature see, e.g., a recent review paper [22].

This paper intends to review the recent development in modeling of coated spherical contact. To obtain physical insights of a coated spherical contact, the modeling work done so far was carried out under some simplifying assumptions such as: the coating is perfectly bonded to the substrate; both the coating and substrate materials are homogeneous and isotropic with zero residual stresses; the sphere bottom is restricted with zero displacement. As shown in Figure 1, the normal loading is achieved by applying a normal contact load *P* or an interference (displacement in the negative *z* direction) *ω* on the rigid flat. The combined normal and tangential loading is achieved by adding a tangential displacement *u*_x_ of the rigid flat upon the completion of the normal loading.

This paper begins with reviewing the modeling work on the coated spherical contact under normal and combined normal and tangential loading (Section 2 and Section 3, respectively). Next, the coated rough surface contact modeling is discussed (Section 4). Then some interesting experimental results are presented, which validate and correlate well with the coated spherical contact modeling (Section 5). The final part of this review is devoted to the modeling of bilayer/multilayer coated spherical contact (Section 6).

## 2. Coated Spherical Contact under Normal Loading

In general, the coating and substrate materials are identified by Young’s moduli *E*_co_, *E*_su_, yield strength *Y*_co_ and *Y*_su_ together with proper hardening rules. The subscripts ‘co’ and ‘su’ indicate the coating and substrate materials, respectively. With increasing normal contact loading, the deformation in the coated sphere is initially elastic until yield inception occurs marking the transition from pure elastic to elastic–plastic deformation. Accordingly, this section consists of three subsections to address pure elastic deformation, yield inception and elastic–plastic deformation in a coated sphere.

### 2.1. Elastic Regime

Keer et al. [23] analytically investigated the elastic normal contact of two identical coated spheres (note that this contact problem is equivalent to the simpler one shown in Figure 1 since the contact interface of two identical coated spheres is also perfectly flat). They examined the effect of the coating thickness on the contact pressure distribution and stress distribution at the coating/substrate interface. This was done for two cases, a deformable coating on a rigid substrate and a stiff coating on a compliant substrate. In the latter case, it was shown that for a given contact radius, the contact pressure increases with increasing coating thickness. The peak normal and shear stresses along the coating/substrate interface first increases then decreases with increasing coating thickness.

To better describe the contact deformation behavior of coated spheres, it is necessary to explore the relations between the various contact parameters such as the interference, contact load and contact area. Goltsberg and Etsion [24,25] investigated these relations using the finite element method. The contact parameters were normalized by their corresponding values, at which the coating and substrate contribute equally to the total normal interference (for more details, see [24]). Such normalization enables the derivation of simple dimensionless universal relations between the various contact parameters. As a result, the dimensional relations can also be easily obtained. Based on the relation between the interference and the contact load [24], an equivalent modulus of elasticity that is load-independent was proposed for the coated spherical contact. This finding differs from the common belief that the equivalent modulus of elasticity of a coated system should vary with the indentation depth as is often reported in the literature, e.g., [9]. This load independent equivalent modulus of elasticity greatly simplifies the analysis of elastic coated spherical contact, which can now become analogous to the well-known Hertz contact solution [24].

### 2.2. Yield Inception

**Figure 2:**
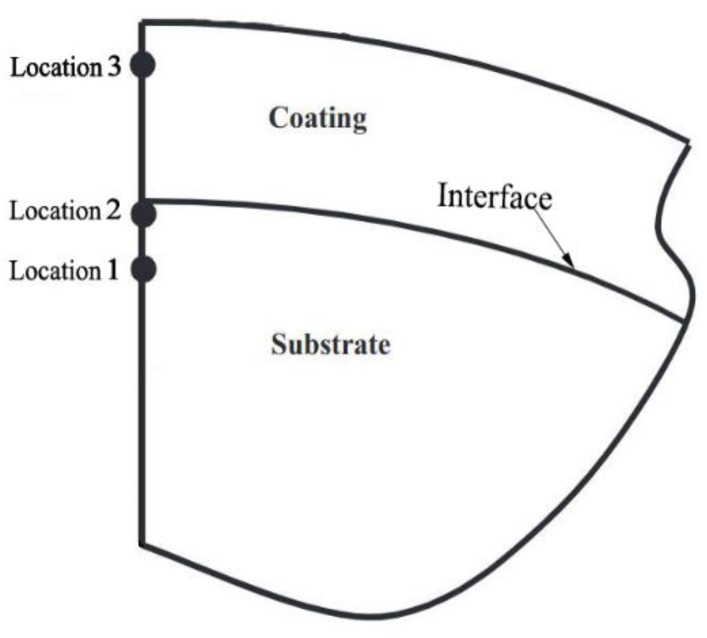
Schematic of the tip of a coated sphere where the three possible locations of yield inception are shown taken from [26].

With increasing contact loading, the stress level in the coated sphere increases and yield inception occurs when the von Mises equivalent stress reaches the yield strength of the relevant material. Goltsberg et al. [26] performed finite element analysis to investigate the effect of the coating thickness and coating and substrate material properties on the yield inception in a coated sphere assuming a slip (frictionless) contact condition. They found that the yield inception always occurs on the axis of symmetry of a coated sphere but can have three possible locations (see Figure 2). The actual location depends on the dimensionless coating thickness *t*/*R* compared to (*t*/*R*)_p_, which is the optimum dimensionless coating thickness for the highest critical contact load, *P*_c_, at yield inception and thus maximum resistance to yield. The value of (*t*/*R*)_p_ depends on the coating and substrate material properties (see Table 1). When *t*/*R* ≥ (*t*/*R*)_p_, the yield inception occurs at location 3 within the coating slightly below the contact interface. When *t*/*R* < (*t*/*R*)_p_, the yield inception occurs at location 2 on the substrate side of the coating/substrate interface. At extremely small *t*/*R*, the yield inception occurs at location 1 within the substrate. Ronen et al. [27] found that the dimensionless coating thickness corresponding to maximum yield resistance under full stick contact condition is similar to that in [26].

The study in [26] covered cases for hard coating on soft substrate materials such that *P*_c_co_ > *P*_c_su_, where *P*_c_co_ and *P*_c_su_ are the critical contact loads at yield inception in a homogeneous sphere made of the coating or substrate material, respectively. It is thus reasonable to expect that the critical load *P*_c_ of a coated sphere at yield inception is at least larger than *P*_c_su_ exhibiting a strengthening effect of hard coating. In fact, near the maximum strengthening at (*t*/*R*)_p_, *P*_c_ can even be larger than *P*_c_co_. However, a surprising weakening effect was also reported in [26] where *P*_c_ of a coated sphere with ultrathin coating is even smaller than *P*_c_su_. In [28], it was found that the weakening effect (*P*_c_ < *P*_c_su_) occurs when *t*/*R* < (*t*/*R*)_T_, which is the transition dimensionless coating thickness, at which the critical contact load *P*_c_ of the coated sphere is equal to *P*_c_su_. This (*t*/*R*)_T_ depends on substrate material properties (see Table 1). Within the range of *t*/*R* < (*t*/*R*)_T_, the coated sphere has the lowest yield resistance at (*t*/*R*)_MW_ exhibiting the maximum weakening effect and (*t*/*R*)_MW_ depends on the coating and substrate material properties (see Table 1). When *t*/*R* is only slightly smaller than (*t*/*R*)_MW_, the yield inception occurs at location 1 within the substrate (see Figure 2).

**Figure 3:**
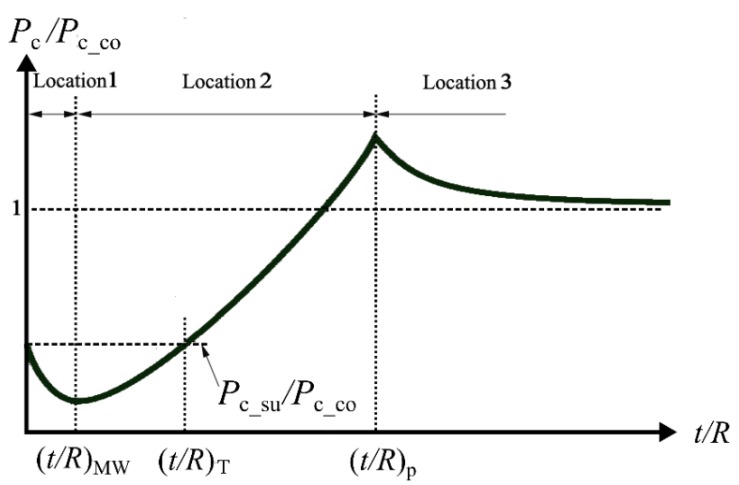
A typical relation between the dimensionless critical contact load *P*_c_/*P*_c_co_ and the dimensionless coating thickness *t*/*R*.

It was shown in [28] that the weakening effect results from additional stresses at the coating/substrate interface due to Young’s modulus mismatch. Such additional stresses escalate the von Mises stress level in the region containing locations 1 and 2 in Figure 2 and lead to premature yield failure. Figure 3 presents a summary of the important results obtained in [26,28]. As would be expected, for very large *t*/*R* the value of *P*_c_ approaches *P*_c_co_ and for *t*/*R =* 0, *P*_c_ = *P*_c_su_. A summary of the physical meaning and empirical expressions of (*t*/*R*)_MW_, (*t*/*R*)_T_ and (*t*/*R*)_p_, which are functions of dimensionless material properties [26,28], is presented in Table 1.

As presented in [29], within the elastic regime, homogenous spherical contacts in both flattening and indentation exhibit identical behavior. This is similar to the case with coated spherical contact [30], where it was shown that the yield resistance behavior of a coated half-space subject to rigid spherical indention is very similar to that shown in Figure 3.

The investigation of yield inception behavior for hard coatings in [26,28,30] was done assuming that *P*_c_co_/*P*_c_su_ > 1. However, this assumption may preclude some realistic cases of hard coatings, which only require that *Y*_co_/*Y*_su_ > 1. The assumption of equal Poisson’s ratios of coating and substrate materials in [26] results in *P*_c_co_/*P*_c_su_ = (*Y*_co_/*Y*_su_)^3^(*E*_co_/*E*_su_)^−2^. Hence for *E*_co_/*E*_su_ = 4 and *Y*_co_/*Y*_su_ = 2 as an example, we have *P*_c_co_/*P*_c_su_ = 0.5 < 1. Therefore, it is necessary to include such cases that were omitted in [26] in order to obtain a more comprehensive understanding of the yield inception behavior of a coated sphere with hard coating.

**Figure 4:**
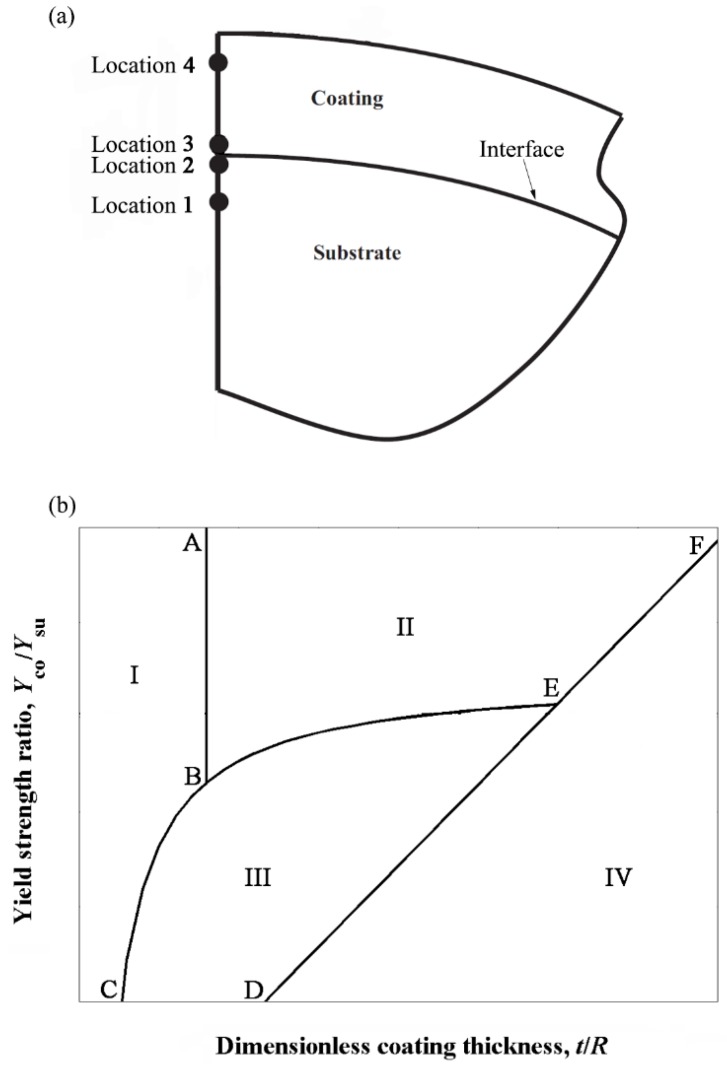
(**a**) Schematic of the tip of a coated sphere where the four possible locations of yield inception are shown and (**b**) a generic yield map presenting the yield inception location as a function of *t*/*R* and *Y*_co_/*Y*_su_, taken from [31].

To this end, Chen et al. [31] considered all the possible cases of hard coatings with *Y*_co_/*Y*_su_ > 1 and *E*_co_/*E*_su_ > 1. In addition to the three possible yield inception locations presented in Figure 2, a new surprising yield inception location was found on the coating side of the coating/substrate interface along the axis of symmetry, see location 3 in Figure 4a, where the locations are renumbered compared to Figure 2. This additional location is typical for relatively small values of *Y*_co_/*Y*_su_ slightly above 1. Thus, the yield inception can occur at one of the four possible locations depending on the dimensionless coating thickness *t*/*R* and dimensionless material properties, which are *Y*_co_/*Y*_su_, *E*_co_/*E*_su_ and *E*_su_/*Y*_su_. Figure 4b demonstrates a generic yield map presenting the yield inception location, for hard coatings, as a function of *t*/*R* and *Y*_co_/*Y*_su_ for given *E*_co_/*E*_su_ and *E*_su_/*Y*_su_. The yield map is divided into four zones I–V corresponding to yield inception locations 1–4 in Figure 4a, respectively. The yield map for a given combination of *E*_co_/*E*_su_ and *E*_su_/*Y*_su_ can be produced by using the empirical expressions of the *t*/*R* values on the zone boundaries, which were also provided in [31]. With the yield map at hand, the yield inception location of any given hard coated spherical contact can be easily predicted. This greatly simplifies and accelerates the procedure of finding a proper coating design where a certain location of yield inception may need to be prevented.

As can be seen from Figure 4b, for cases with *Y*_co_/*Y*_su_ larger than that at point E, the yield inception location changes from location 1 to 2 to 4 with increasing *t*/*R* in the same way as shown in Figure 3 (note that in Figure 3 the locations are numbered in a different order from Figure 4a). Hence, (*t*/*R*)_AB_ = (*t*/*R*)_MW_ and (*t*/*R*)_DEF_ = (*t*/*R*)_p_ (see Table 1). However, cases with *Y*_co_/*Y*_su_ smaller than that at point E were not studied in [31]. To bridge this gap, future investigation of yield resistance vs. *t*/*R* (similar to Figure 3) is required for the cases of *Y*_co_/*Y*_su_ values smaller than that at point E in Figure 4b. Table 2 summarizes the physical meaning of the *t*/*R* values of the various zone boundaries of the yield map in Figure 4b. Note that the still-missing cases are marked in the table as not reported.

The aforementioned studies [26,27,28,30,31] offered useful insights into the yield inception behavior of a coated spherical contact for hard coatings. Despite the weakening effect that occurs in a narrow range of extremely small coating thicknesses [28], the hard coating increases the yield resistance of a coated system. This, in general, agrees with our common sense that a hard coating should be helpful to protect a soft substrate. In light of this, not enough research effort was devoted so far to investigating yield inception behavior in the case of soft coatings.

Song et al. [32] analyzed the elastic indentation of a coated half-space and reported that soft coatings, unlike hard coatings, weaken the yield resistance of a coated system in the range of moderate coating thicknesses. A similar finding was also reported by Zhao et al. [33] for the elastic–plastic flattening of a coated sphere. However, the yield resistance behavior for ultra-thin coatings was not addressed in [32,33]. Goltsberg et al. [34] analyzed the flattening case of soft coatings and found that the yield resistance vs. the coating thickness for the whole range of coating thicknesses is a mirror image of that for hard coatings shown in Figure 3. In the range of moderate *t*/*R*, there exists a (*t*/*R*)_m_, which corresponds to maximum weakening (instead of maximum strengthening at (*t*/*R*)_p_ in hard coating as shown in Figure 3). For ultra-thin coatings, the yield resistance of a coated sphere with a soft coating is higher than that of a homogeneous sphere made of the harder substrate material. This indicates that the ultra-thin soft coating strengthens the coated system, which is opposite to the weakening effect of ultra-thin hard coatings. In soft coatings there is a certain *t*/*R* corresponding to the maximum strengthening effect that is opposite to (*t*/*R*)_MW_ in Figure 3. However, a more comprehensive study is needed to further explain such an interesting strengthening effect of soft coatings for a wider range of material properties, which is missing in [34].

### 2.3. Elastic–Plastic Regime

Purely elastic contact deformation in a coated sphere may be desired to avoid residual plastic deformation after unloading. However, this ideal goal is usually difficult to achieve in practice. On the other hand, the elastic–plastic contact deformation can be advantageous in some cases. For instance, to obtain high electrical contact conductance it is favorable to have a large contact area, typical to large elastic–plastic deformation. Therefore, it is of practical importance to investigate the elastic–plastic coated spherical contact.

Eid et al. [35] simulated the contact between MEMS switches through modeling the elastic–plastic contact between a rigid flat and a soft gold (Au) sphere with thin hard ruthenium (Ru) coating in the presence of adhesion. Two thicknesses were considered. They found that for a given contact load the coated sphere with thinner coating has a larger contact area. This increases the contact conductance but also undesirably increases the pull-off force due to adhesion. The authors thus suggested future research for seeking a proper coating material and thickness to obtain a sufficiently large contact area but with an acceptable pull-off force. Therefore, to establish a generic guideline for the coating selection, it is imperative to take account of a large range of coating thicknesses and material properties.

**Figure 5:**
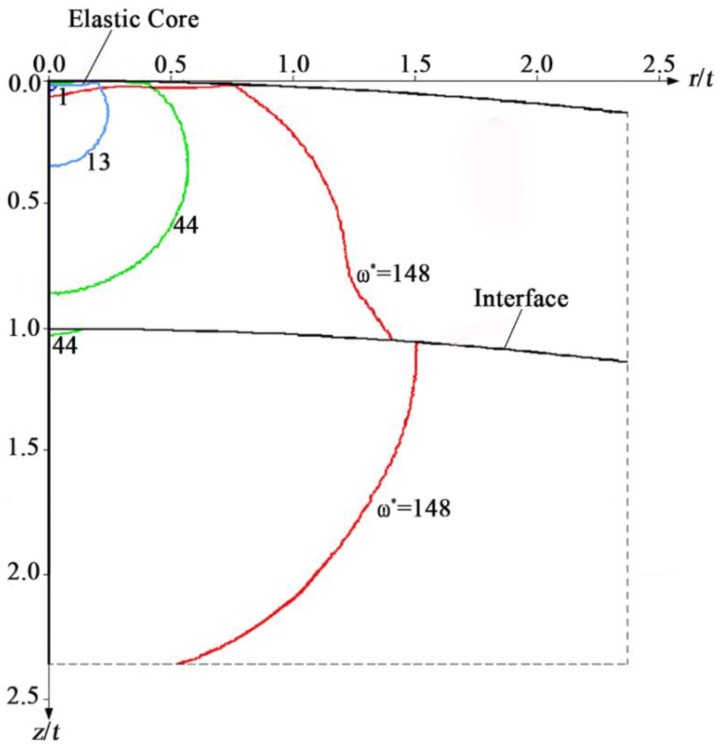
The evolution of plastic zones in a coated sphere with increasing interference *ω∗*, taken from [36].

Chen et al. [36] investigated the evolution of plastic zones in a hard coated sphere in contact with a rigid flat under slip (frictionless) contact condition covering a large range of moderate thicknesses and material properties. Moderate thicknesses were selected to avoid the weakening effect associated with undesirable yield inception locations [28]. Figure 5 demonstrates the evolution of plastic zones in a coated sphere with increasing contact loading (represented by dimensionless interference ω*). The results in Figure 5 are for a typical case of *t*/*R* = 0.05, *E*_co_/*E*_su_ = 2 and *E*_co_/*Y*_co_ = *E*_su_/*Y*_su_ = 1000. Due to the axisymmetry, only the symmetry plane is demonstrated extending to 2.5*t* in both radial and axial directions from the sphere summit. Plastic zones corresponding to several values of the dimensionless interference *ω*^∗^ are presented. ω* = *ω*/*ω*_c1_, where *ω*_c1_ is the first critical interference at the first yield inception in the coated sphere. *ω*_c1_ depends on the coating thickness and material properties and is slightly larger than *ω*_c_co_, which is the critical interference of a homogenous sphere made of the coating material. The plasticity initiates within the coating (first yield inception) slightly below the contact interface on the axis of symmetry. With increasing interference, a plastic zone forms and expands in the coating. Subsequently, plasticity initiates on the substrate side of the coating/substrate interface (second yield inception) on the axis of symmetry and a plastic zone forms and expands in the substrate.

An interesting finding is that an elastic core (see Figure 5) exists between the plastic zone in the coating and the contact interface. Following the second yield inception in the substrate, the size of this elastic core increases with increasing interference because the accumulated plasticity in the softer substrate relieves the stress level in the coating. It was found in [36] that with increasing normal loading the coating thickness along the axis of symmetry decreases. However, due to the stress relief in the coating following the second yield inception in the substrate, this thickness reduction is slower and, for cases of relatively high *E*_co_/*E*_su_ and small *t*/*R*, the coating thickness eventually recovers. Moreover, the mean contact pressure reaches a maximum at about the interference corresponding to the second yield inception in the substrate and thereafter decreases with increasing interference.

Later, careful scrutiny by Chen et al. [37] showed that the maximum mean contact pressure is actually at an interference slightly larger than that at the second yield inception in the substrate. Thereafter, with increasing interference the mean contact pressure decreases from its maximum and the rate of decrease slows down with further interference increase. When *ω* is larger than a certain value, the decreasing rate becomes negligibly small and the mean contact pressure becomes constant, defining the effective hardness of the coated sphere. The various contact parameters (contact load, contact area and interference) were normalized by their corresponding values at the maximum mean contact pressure leading to simple dimensionless relations between the contact parameters. Consequently, the dimensional relations were easily obtained.

Note that the studies in [35,36,37] were all carried out under slip contact condition. Ronen et al. [27] compared the elastic–plastic coated spherical contact under stick and slip conditions. They found that the contact condition has a negligible effect on the plasticity evolution in a coated sphere except in the region close to the contact interface. Likewise, they normalized the contact parameters by their corresponding values at the second yield inception in the substrate and provided simple dimensionless relations between the contact parameters up to the second yield inception.

A common feature of the studies in [27,35,36,37] is that the elastic–plastic coating and substrate materials exhibit a linear hardening behavior with a tangent modulus of 2% of Young’s modulus. However, Lu et al. [38] pointed out that the power-law hardening may be more appropriate to describe the behavior of coated spheres. Moreover, for hard coating/soft substrate system, the coating is only elastically deformed in some practical applications such as the journal bearings in marine engines. Hence, they used elastic coating material and elastic–plastic substrate material with power-law hardening for the coated sphere and examined the effect of the hardening exponent of the substrate material on the loading [38] and unloading [39] contact behavior. For the case of loading, to a given interference, as the hardening effect becomes stronger the contact load increases and the contact area decreases. In addition, the coating thickness along the axis of symmetry decreases with increasing interference at a higher rate for a stronger hardening effect. For the case of unloading from a given maximum interference, as the hardening effect becomes stronger the residual interference decreases and hence, the energy loss due to plastic deformation decreases. The study of elastic–plastic contact behavior subjected to cyclic loading–unloading, which commonly exists in micro-switch applications, is still missing in the published literature. Under cyclic loading–unloading, the shakedown phenomenon may be observed, similar to the homogenous spherical contact [40]. Thus, it is strongly recommended to investigate in the future the potential shakedown phenomenon in the coated spherical contact.

As mentioned earlier, the literature on coated spherical contact with soft coatings is relatively sparse. Zhao et al. [33] investigated such a problem for one loading cycle, where both coating and substrate materials are elastic–plastic with power-law hardening. For fixed substrate material properties, at a given interference, the contact area increases significantly with decreasing hardening exponent of the coating material but is negligibly affected by coating thickness or other coating material properties. In addition, they also suggested further investigation of the effect of substrate material properties.

## 3. Coated Spherical Contact under Combined Normal and Tangential Loading

To study the friction mechanism of coated surfaces, it is necessary to consider the coated spherical contact under combined normal and tangential loading. Keer et al. [23] investigated the elastic contact of two identical coated spheres under such combined loading. They assumed that slip can occur locally where Coulomb friction law is satisfied at the originally stick contact interface. As the tangential loading increases, slip initiates at the contact boundary forming an annular slip zone. Thereafter, this annular slip zone progresses radially inward leading to slip across the entire contact interface. The integral of the corresponding shear traction over the contact interface is thus the static friction force, which solely depends on the normal load and the empirical Coulomb friction coefficient.

In practice, the contact deformation is not necessarily elastic but often elastic–plastic. This calls for the need to study the elastic–plastic coated spherical contact under combined loading. Moreover, mounting evidence suggested that the empirical Coulomb friction law may not be appropriate to describe the complex interfacial friction behavior, which results from various intertwined physical and chemical sources [41]. An alternative and simple approach is to assume that the contact interface maintains full stick throughout the loading process. This was validated experimentally [42] for copper spheres under combined normal and tangential loading by a hard sapphire flat. It also justified the assumption of full stick contact condition made in [20] for modeling combined loading of homogeneous spherical contact. Hence, the assumption that the contact interface maintains full stick seems to be valid and greatly simplifies the problem. Therefore, the modeling work on the coated spherical contact under combined loading presented in [43,44,45] considered elastic–plastic deformation and adopted the full stick assumption.

Chen and Etsion [43] investigated this contact problem using a coated sphere in contact with a rigid flat (see Figure 1) for the case of hard coatings *Y*_co_/*Y*_su_ > 1 (assuming *E*_co_/*E*_su_ > 1). Chen [44] and Zhang et al. [45] investigated the case of soft coatings *Y*_co_/*Y*_su_ < 1 (assuming *E*_co_/*E*_su_ < 1). In [43,44,45], a normal load *P* was first applied so that the coated sphere deforms elastic–plastically. Subsequently, a tangential displacement *u*_x_ was applied and increased incrementally. The corresponding tangential reaction force was obtained as the tangential contact load *Q*. It was found that the tangential stiffness of the contact junction ∂*Q*/∂*u*_x_ decreases with increasing *u*_x_ due to the accumulated plasticity in the contact junction. Sliding inception was assumed when ∂*Q*/∂*u*_x_ decreases to a small value that is less than 10% of its initial value. Hence, the tangential contact load at sliding inception is considered as the static friction force. Accordingly, the static friction coefficient *μ* can be easily calculated.

**Figure 6:**
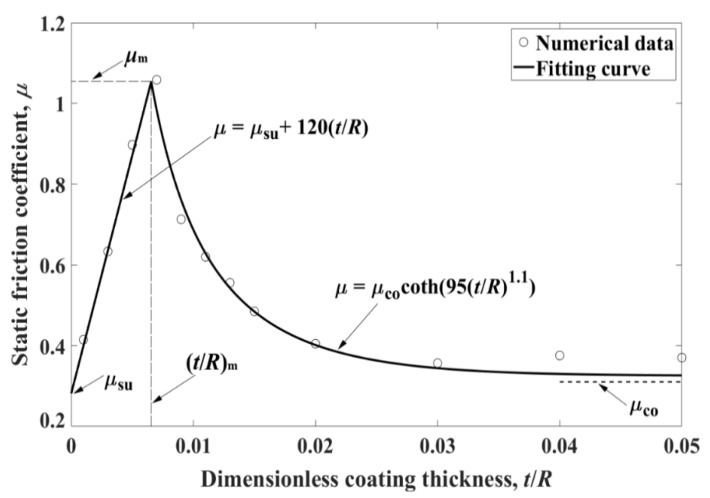
Typical behavior of the static friction coefficient *μ* as a function of *t*/*R*, taken from [43].

Figure 6 presents the static friction coefficient *μ* as a function of the dimensionless coating thickness *t*/*R* for a typical case of *E*_co_/*E*_su_ = 4, *E*_co_/*Y*_co_ = *E*_su_/*Y*_su_ = 1000 and *P*/(*E*_su_*R*^2^ × 10^−7^) = 50. The values of *μ*_su_ and *μ*_co_ are the static friction coefficients obtained for homogeneous spheres made of the substrate and coating materials, respectively. With increasing *t*/*R*, the static friction coefficient *μ* first increases from *μ*_su_ reaching a maximum at *t*/*R* = (*t*/*R*)_m_ and then decreases approaching *μ*_co._ Such *μ* behavior with increasing *t*/*R* was attributed to the competition of two mechanisms [43]. One is the decreasing plastic volume in the substrate with increasing *t*/*R*, which results in a higher tangential stiffness of the contact junction and hence, increasing *t*/*R*. The other mechanism is the decreasing contact area with increasing *t*/*R*, which results in a contact junction less capable of supporting the tangential force and hence, decreasing *μ*. The first mechanism dominates in the range of relatively small *t*/*R*, while the second one dominates in the range of relatively large *t*/*R*. Therefore, *μ* exhibits friction behavior as shown in Figure 6.

It was demonstrated in [34] that soft coatings exhibit a mirror image behavior of hard coatings in terms of the yield resistance. For the static friction coefficient, Chen [44] and Zhang et al. [45] showed that the soft coatings also exhibit a mirror image behavior of hard coating (Figure 6). Hence, opposite to Figure 6, in the case of soft coatings, there exists a (*t*/*R*)_m_ corresponding to the lowest *μ* For *t*/*R* < (*t*/*R*)_m_, *μ* decreases with increasing *t*/*R*. For *t*/*R* > (*t*/*R*)_m_, *μ* increases with increasing *t*/*R*. Similar effect of coating thickness on surface friction has been reported in experimental studies for indium coated tool steel surface [46] and silver-coated hardened M2 steel surface [47]. The *μ* behavior as a function of *t*/*R* in [44,45] is explained in [45] by the competition of two mechanisms. One is that a thicker soft coating decreases the tangential stiffness of the contact junction leading to decreasing *μ*. The other is that increasing contact area with increasing *t*/*R* results in a contact junction capable of supporting larger tangential force and hence, increasing *μ*. Empirical expressions for the static friction coefficient *μ* that cover a large range of coating thicknesses, material properties, and normal loading were provided for the case of hard [43] and soft coatings [45]. Table 3 summarizes the information regarding the most important (*t*/*R*)_m_, where (*t*/*R*)_m_ depends on *E*_su_/*Y*_su_ and the dimensionless normal load P* *= P*/(*E*_su_*R*^2^ × 10^−7^). It should be noted that the work in [43,45] can be further expanded to investigate the coated spherical contact under cyclic tangential loading to explore the potential shakedown phenomenon and fretting wear behavior similar to the case of uncoated spherical contact [48].

## 4. Coated Rough Surface Contact

**Figure 7:**
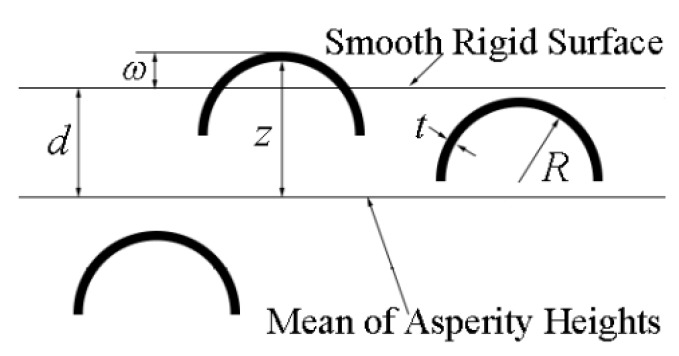
A model of a coated rough surface in contact with a smooth rigid surface, taken from [49].

Chen and Etsion [49] incorporated the contact behavior of a single coated asperity [24,25,37] in a statistical coated rough surface model, shown schematically in Figure 7, to analyze the coated surface contact. In the figure, *z*, *d* and *ω* are the asperity height measured from the mean of the asperity heights, the surface separation and the interference of a contacting coated asperity, respectively. The coated rough surface is modeled by a substrate surface based on the GW model [8] and a coating that replicates the topography of the substrate surface. Hence, the roughness of the coated surface stems from the substrate surface. In other modeling work, e.g., [50,51], the roughness of the coated surface is assumed as entirely contributed by the coating surface while the substrate surface is perfectly smooth. A significant disadvantage of this modeling approach is that it fails to consider the effect of the substrate surface roughness, which may affect friction and wear properties of coated surfaces [52,53]. In [52] for coating thickness from 0.1 to 1 m it was shown that the difference in surface roughness before and after coating is less than 20% and hence, can be ignored. This in fact validates the above assumption that was made in [49] regarding coating that replicates the topography of the substrate surface.

In the modeling approach used in [49], the coated rough surface comprises many identical coated asperities (see Figure 7) with heights that vary statistically following a Gaussian distribution. Hence, the total surface contact load and contact area under a certain surface separation can be obtained by summing these values of each contacting coated asperity. In [49], only the normal contact behavior of the coated surface contact was investigated through a parametric study. It was found that a coated surface with a thicker, stiffer and harder coating and a rougher substrate, under a given contact load, has a higher surface separation and smaller contact area.

Note that in [49] the contact of two coated rough surfaces was simplified as an equivalent rough surface in contact with a rigid flat. To examine whether this simplification affects the physical insights reported in [49], Gu and Wang [54] developed a contact model of two coated rough surfaces based on the approach proposed by Greenwood and Tripp [55]. This model enabled them to consider the effect of the asperity misalignment. However, their results showed close agreement with those in [49]. Hence, it demonstrates that the effect of the asperity misalignment is marginal and it is sufficient to model the coated rough surface contact using an equivalent rough surface in contact with a rigid flat.

Unfortunately, the friction behavior of coated surfaces under combined normal and tangential loading has not yet been investigated. Since it was demonstrated that soft coatings can reduce the static friction at an asperity level [44,45], it is highly worth future research to explore whether the soft coatings can also reduce the static friction at a rough surface level.

## 5. Experimental Validation and Correlation

**Figure 8:**
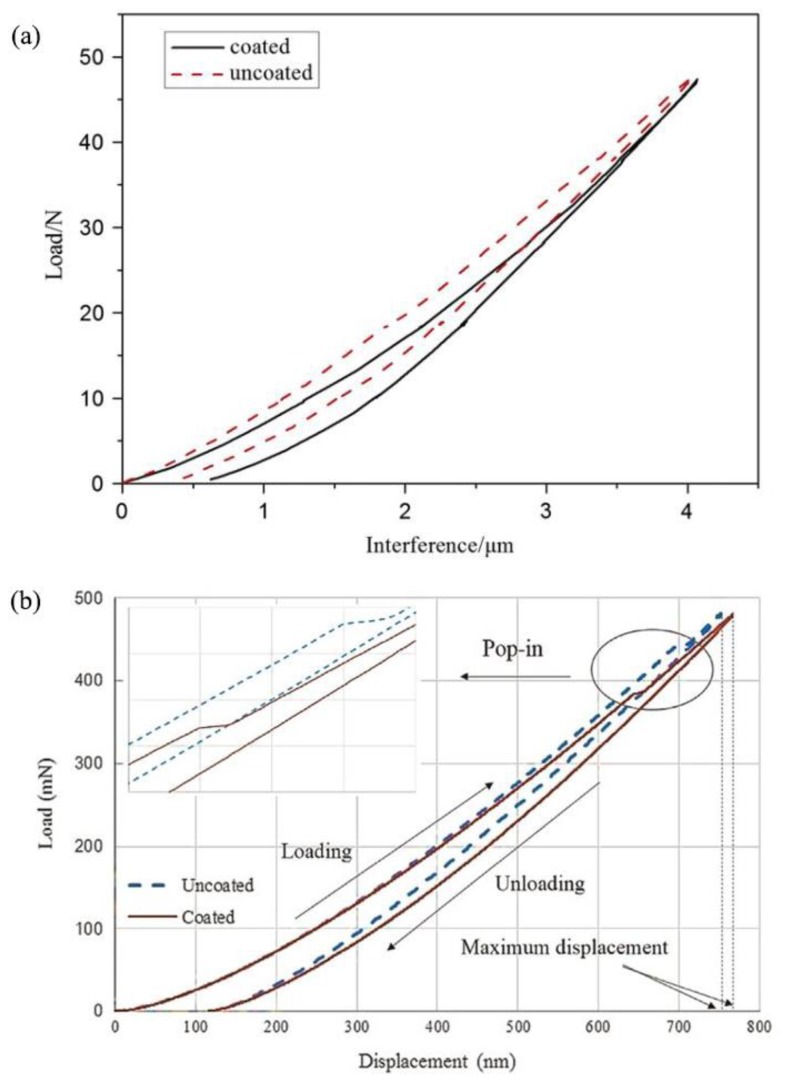
(**a**) Typical load–interference relation obtained from flattening of a coated and an uncoated sphere (taken from [56]) and (**b**) typical load–displacement relation obtained from the indentation of coated and uncoated silicon flat (taken from [57]).

Since the yield resistance behavior of a coated spherical contact subject to indentation or flattening is very similar [30], the theoretical results regarding the weakening effect [26,28] were validated experimentally in both cases of flattening [56] and indentation [57]. In [56], the yield resistance was implicitly obtained as the amount of plastic deformation in the coated sphere that was quantified by the residual interference after one loading–unloading cycle with a fixed maximum load. A higher residual interference indicates more plastic deformation due to a lower yield resistance. Figure 8a presents a typical load–interference relation obtained from flattening coated and uncoated brass spheres with radius of 2.25 mm. The coating is TiN with thickness of 2.3 m. As can be seen, the coated sphere has a higher residual interference and thus a lower yield resistance.

In [57], the critical contact load at yield inception was explicitly obtained as the indentation load corresponding to the appearance of pop-in (discontinuity) in the indentation load–displacement curve. This is because single-crystalline silicon was used as the substrate material. The yield inception in this material is caused by phase transformation characterized by the pop-in behavior. Figure 8b presents a typical load-displacement relation obtained from the indentation of coated and uncoated silicon flat by spherical diamond indentor with tip radius of 20 m. The coating is Tungsten with a thickness of 700 nm. As can be seen, the coated flat exhibits pop-in at a lower load than the uncoated case and thus has a lower yield resistance. Therefore, these two experimental studies [56,57] validate the weakening effect of thin hard coatings [28]. Moreover, it was suggested in these studies that the weakening effect could be alleviated by using coating and substrate materials with a smaller Young’s modulus mismatch. This is in line with the theoretical results in [28].

Bar-Hen and Etsion [58] conducted an experimental study to examine the effect of coating thickness *t* and substrate surface roughness *R*_su_ (the mean radius of curvature of substrate asperities) on the flank wear of coated cutting tools, where the coating/substrate system is TiAlN/Tungsten carbide (WC). Fifty tools with various values of *t* and *R*_su_ were tested under the same working conditions for the same length of turning time. The various *t* values were obtained by placing the cutting tools on several racks with different spacing in the coating chamber. The various *R*_su_ values were obtained by using a brushing–polishing machine to polish the tool flank surface prior to coating with abrasive polishing paste of different grain sizes.

**Figure 9:**
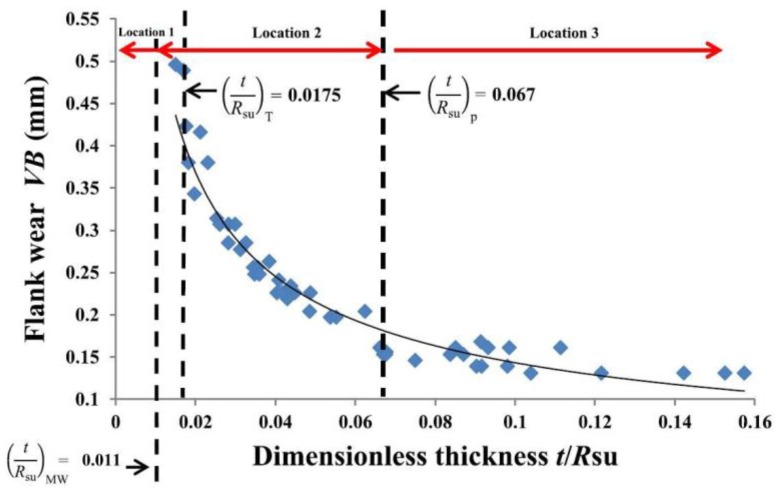
Flank wear vs. dimensionless thickness *t*/*R*_su_ with the range of values corresponding to yield inception location in a coated asperity as shown in Figure 2 (taken from [58]).

The flank wear after testing was measured and plotted against the coating thickness *t*, substrate surface roughness *R*_su_ and their ratio *t*/*R*_su_. A general trend in the three plots is that the flank wear decreases with increasing *t*, decreasing *R*_su_ and increasing *t*/*R*_su_. However, the correlation between the flank wear and *t*/*R*_su_ has 94% goodness of fit (see Figure 9), which is much better than that for the coating thickness alone (57%) or the substrate roughness alone (63%). This strong dependence on *t*/*R*_su_ indicates that *t*/*R*_su_ is indeed a key parameter to characterize wear resistance of coated cutting tools.

The theoretical results in [26,28] (see Figure 3) also show that the yield resistance and yield inception location depend on *t*/*R*. The values of (*t*/*R*_su_)_MW_, (*t*/*R*_su_)_T_ and (*t*/*R*_su_)_P_ shown in Figure 9, were calculated based on the material properties of TiAlN/WC using the relevant empirical expressions provided in Table 1. The coated cutting tools experience higher wear when the yield inception of a coated asperity is at location 1 or 2 namely, within the substrate or on the substrate side of coating/substrate interface (see Figure 2). The wear in the coated cutting tools becomes much milder when *t*/*R*_su_ is larger than (*t*/*R*_su_)_P_ with the yield inception at location 3 within the coating (see Figure 2). Hence, the dependence on *t*/*R* in both theoretical results [26,28] and experimental results [58] implies that *t*/*R*, which combines both the coating thickness and substrate roughness, is an important parameter in coated rough surface modeling and in the coating design. It also suggests that the yield resistance or yield inception location might be physically related to the wear resistance of the coated system, which is worth exploring in the future.

In order to minimize wear of coated cutting tools, it is recommended in [58] that *t*/*R*_su_ of the coated surface should be 50% larger than (*t*/*R*_su_)_P_. In practical coating design of some real application, this can be easily achieved by controlling the coating deposition parameters to obtain the desired coating thickness for a given substrate topography.

It should be noted here that in some prior studies, e.g., [59,60], the coating thickness was normalized by the Hertzian contact radius in the absence of coating. However, since the Hertzian contact radius depends on the normal load, this normalization does not seem to be very convenient to evaluate the dimensional coating thickness of an actual coated system. On the other hand, with *t*/*R*, the coating thickness is normalized by the mean radius of curvature of substrate asperities, which conveys more straightforward physical meaning. Based on the interesting experimental validation and correlation obtained with *t*/*R* [56,57,58], this dimensionless form for the coating thickness is recommended in the coating design and research.

## 6. Bilayer/Multilayer Coated Spherical Contact

All the above reviewed work focused on single-layer coated spherical contact. A hard coating layer can enhance the wear properties of the coated system [61,62] while a soft coating layer can be used as solid lubricant [7,63] and reduce friction. Nonetheless, in some scenarios, multiple properties such as strong yield resistance, low surface adhesion and high electrical conductivity are desired simultaneously. This calls for bilayer/multilayer coatings. Despite a large volume of literature on the indentation of bilayer/multilayer coated surfaces [64,65,66,67,68], little attention has been paid so far to the flattening of bilayer/multilayer coated asperities where abrasive wear can be eliminated.

Some limited work was reported on the flattened bilayer coated spherical contact [69,70]. Korchevnik et al. [69] investigated the electrical contact resistance (ECR) of a bilayer coated sphere consisting of a hard outer coating and soft interlayer compared to the substrate material. This bilayer coating design was selected because the hard, outer coating, can increase the durability of the electrical junction under mechanical contact loading and the soft interlayer can enlarge the contact area to decrease the ECR. Only elastic contact behavior was studied in [69]. To increase the contact area for a lower ECR, the interlayer material of lower Young’s modulus and smaller Poisson’s ratio should be used. This work is a first attempt to model the ECR of a bilayer coated spherical contact. More work can be done in the future to consider the effect of elastic–plastic deformation and the ECR model of a bilayer coated rough surface.

It was found in [28] that Young’s modulus mismatch at the coating/substrate interface can escalate the stress level and lead to premature yield failure in a coated sphere. Thus, inserting an interlayer with an intermediate Young’s modulus can be a solution to avoid this undesirable phenomenon. Indeed, Parel et al. [70] found that a bilayer coated sphere has a lower stress level at the material interface compared to a single layer case. This results in a stronger yield resistance. However, a few exceptional cases were also reported in [70] where the yield resistance becomes weaker. Such cases are those where the total dimensionless coating thickness *t*/*R* (the sum of thicknesses of the bilayers normalized by the substrate radius) is below a certain value, which depends on the material properties and the dimensionless interlayer thickness *t*_1_/*t*.

The finding of exceptional cases in [70] is not surprising since the abrupt change of material properties at the material interface still exists in the bilayer coated sphere. To avoid this disadvantage of the bilayer coating, the multilayer coating may be used. Recently, Chen and Yue [71] investigated the contact behavior of a multilayer coated sphere. However, this analysis is limited to elastic regime and the information regarding the yield resistance is not provided. Therefore, it is highly recommended to expand the work in [70] to explore the yield resistance behavior of a multilayer coated sphere.

## 7. Conclusions

The coated spherical contact modeling, based on a coated sphere flattened by a rigid flat, was reviewed. For the coated spherical contact under normal loading, important results were summarized for the elastic deformation regime, yield inception and elastic–plastic deformation regime. For the coated spherical contact under combined normal and tangential loading, the existing work mainly focused on the static friction at the asperity contact level. An interesting finding is that the hard and soft coatings exhibit mirror image behaviors in terms of the yield resistance and static friction coefficient, which are functions of the dimensionless coating thickness *t*/*R*. The coated rough surface contact modeling that incorporates the contact behavior of a single coated asperity was introduced. In addition, a brief discussion was devoted to some experimental results that showed an interesting correlation with the theoretical modeling results of a coated spherical contact. Finally, some limited work on the bilayer/multilayer coated spherical contact was discussed.

Future work on various important aspects was suggested, e.g., seeking physical explanation for the strengthening effect of soft coatings, exploring the potential shakedown phenomenon in a coated spherical contact subjected to cyclic loading–unloading, whether soft coatings can reduce the surface friction, whether yield resistance and yield inception location of a coated asperity is physically related to the wear resistance of coated systems and further investigation on the bilayer/multilayer coated spherical contact. It should be noted that all the modeling work in this review employed material behaviors that are characterized by phenomenological constitutive laws. This ignores microstructure effect on the material behavior, which can be crucial if the coated sphere has dimensions close to a material grain size. Moreover, the assumption that the coating replicates the substrate surface topography may become invalid when the coating is sufficiently thick and its own topography cannot be ignored. Therefore, future research to overcome these limitations is recommended.

## Figures and Tables

**Table 1:** A summary of physical meaning and empirical expressions of (*t*/*R*)_MW_, (*t*/*R*)_T_ and (*t*/*R*)_T_ in [26,28].

Item	(*t*/*R*)_MW_	(*t*/*R*)_T_	**(*t*/*R*)_p_**
Physical meaning	Lowest yield resistance	Transition from weakening to strengthening effect	Highest yield resistance
Empirical expression	$1.066{\left(E_{\mathrm{su}}/Y_{\mathrm{su}}\right)}^{-1}{\left(E_{\mathrm{co}}/E_{\mathrm{su}}\right)}^{-0.225}$	$1.417{\left(E_{\mathrm{su}}/Y_{\mathrm{su}}\right)}^{-0.959}$	$2.824{\left(E_{\mathrm{co}}/E_{\mathrm{su}}\right)}^{0.536}{\left(E_{\mathrm{co}}/Y_{\mathrm{co}}\right)}^{-1.608}{\left(E_{\mathrm{su}}/Y_{\mathrm{su}}\right)}^{0.594}$

**Table 2:** Physical meaning of *t*/*R* values on zone boundaries in the yield map.

*Y*_co_/*Y*_su_	(*t*/*R*)_AB_	(*t*/*R*)_DEF_	(*t*/*R*)_CBE_
Above Point E	(*t*/*R*)_MW_, lowest yield resistance	(*t*/*R*)_p_, highest yield resistance	Not exist
Below Point E	Not reported	Not reported	Not reported

**Table 3:** A summary of physical meaning and empirical expressions of (*t*/*R*)_m_ for hard [43] and soft coatings [45].

Item	Hard Coatings	Soft Coatings
Physical meaning of (*t*/*R*)_m_	Highest *μ*	Lowest *μ*
Empirical expression of (*t*/*R*)_m_	$7.59\times 10^{-5}{\left(P^{*}\right)}^{0.493}{\left(E_{\mathrm{su}}/Y_{\mathrm{su}}\right)}^{0.364}$	$3.65\times 10^{-6}{\left(P^{*}\right)}^{0.433}{\left(E_{\mathrm{su}}/Y_{\mathrm{su}}\right)}^{0.648}$
