# Erratum: Chen, Z. and Etsion, I. Recent Development in Modeling of Coated Spherical Contact. *Materials* 2020, *13*, 460

**DOI:** 10.3390/ma13153362

**Published:** 2020-07-29

**Authors:** Zhou Chen, Izhak Etsion

**Affiliations:** Department of Mechanical Engineering, Technion-Israel Institute of Technology, 32000 Haifa, Israel; chenzhouhit@gmail.com

The authors wish to make the following corrections to this paper [1]:

References

45. Zhang, H.; Chen, Z.; Etsion, I. Model for the static friction coefficient of spherical contact with soft coating. *Tribol. Lett*. under review.
70. Parel, K.; Chen, Z.; Etsion, I. Strengthening and weakening effects in bilayer coated spherical contact. *Front. Mech. Eng.* under review.

to the correct version, as follows:

45. Zhang, H.; Chen, Z.; Etsion, I. Model for the static friction coefficient of spherical contact with soft coating. *SN Appl. Sci*. **2020**, *2*, 1197, doi:10.1007/s42452-020-2995-6.
70. Parel, K.; Chen, Z.; Etsion, I. Strengthening and weakening effects in bilayer coated spherical contact. *Front. Mech. Eng.* **2020**, *6*, 23, doi:10.3389/fmech.2020.00023.

The authors would like to apologize for any inconvenience caused to the readers by these changes.
